# The relationship between self-reported habitual exercise and visual field defect progression: a retrospective cohort study

**DOI:** 10.1186/s12886-016-0326-x

**Published:** 2016-08-23

**Authors:** Satoshi Yokota, Yuji Takihara, Kanako Kimura, Yoshihiro Takamura, Masaru Inatani

**Affiliations:** 1 Department of Ophthalmology, Faculty of Medical Sciences, University of Fukui, Japan, 23-3 Matsuokashimoaizuki, Eiheiji, Yoshida, Fukui 910-1193 Japan; 2 Department of Ophthalmology and Visual Sciences, Kyoto University Graduate School of Medicine, Japan, Yoshidakonoecho, Sakyo, Kyoto, Kyoto 606-8501 Japan

**Keywords:** Self-reported habitual exercise, Glaucoma, Visual field defect

## Abstract

**Background:**

Exercise reduces intraocular pressure (IOP) in the short term. However, it is not known whether exercise contributes to slower glaucomatous visual field defect progression.

**Methods:**

Twenty-four primary open-angle glaucoma or exfoliation glaucoma patients who were evaluated by the Humphrey Field Analyzer (HFA) 24–2 program ≥ four times in 3 years were enrolled. Patients with a history of intraocular surgery in past 3 years or other eye diseases threatening visual fields were excluded. Patients were classified into two groups whether they had exercise habits or not.

**Results:**

Eleven patients had exercise habits. The mean ± standard error of IOP and MD slope were 14.8 ± 0.9 mmHg and +0.20 ± 0.20 dB/year in the exercise group and 13.3 ± 0.8 mmHg and −0.53 ± 0.18 dB/year in the non-exercise group (*P* = 0.24 and *P* = 0.01, respectively). Higher IOP [odds ratio (OR) = 0.44/1 mmHg increase; *P* = 0.02] and habitual exercise (OR = 0.04; *P* = 0.02) reduced the visual field defect progression risk in logistic regression analyses.

**Conclusions:**

Patients with self-reported exercise habits had slower glaucoma progression.

**Electronic supplementary material:**

The online version of this article (doi:10.1186/s12886-016-0326-x) contains supplementary material, which is available to authorized users.

## Background

Pathogenesis of glaucoma, the leading cause of irreversible blindness worldwide, is related to intraocular pressure (IOP) [1]. Systemic factors—blood flow, intracranial pressure, and migraine—and lifestyle or environmental factors are associated with primary open-angle glaucoma [2]. Smoking and occupational exposure to pesticides increases glaucoma risk; whereas consumption of fruits and vegetables and omega-3 fatty acid are associated with lower glaucoma risk [2, 3].

Although exercise lowers IOP, the exact mechanism remains unclear [4]. Exercise intensity is related to glaucoma prevalence. For example, faster 10-km race performance predicted lower glaucoma risk [5]. However, the relationship between exercise and visual field defect progression remains unknown. We examined the relationship between self-reported habitual exercise and visual field defect progression to determine exercise use in glaucoma patients.

## Methods

### Patient selection

All patients visited the Department of Ophthalmology, University of Fukui Hospital, between August 2014 and March 2015 and met all inclusion criteria:

1. Primary open-angle glaucoma or exfoliation glaucoma.
2. Four or more visual field tests using the Humphrey Field Analyzer (HFA) Swedish interactive thresholding algorithm (SITA) standard 24–2 program (Carl-Zeiss Meditec Co Ltd, Tokyo, Japan) during the previous 3 years.

Exclusion criteria:

1. Eyes that underwent ocular surgery, including anti-vascular endothelial growth factor injection during the previous 3 years.
2. Eyes with other ocular diseases influencing the perimetry in the short-term (e.g., vitreous hemorrhage and corneal ulcer).

If both eyes met the criteria, one eye was randomly selected using a random number table.

### Self-reported exercise habits

Patients were classified into two groups by a single self-reporting questionnaire: “Do you have habitual exercise more than 30 min per week?” Patients were classified into the exercise and non-exercise group based on their answers.

### Patient data extraction

Patient data were retrospectively reviewed from their clinical records. Mean deviation (MD) of HFA, IOP, and decimal visual acuity were extracted from their clinical records.

### Visual field testing

SITA standard 24–2 perimetry program with HFA was used for visual field testing. Reliable tests were defined as those with < 30 % fixation losses and false-positive or -negative responses. From the result of visual field test, MD slope was calculated by HfaFiles (Beeline Co Ltd, Tokyo, Japan). Visual filed defect progression was defined as MD slope worse than −0.50 dB/year [6].

### Primary and secondary outcome

Primary outcome was the comparison of MD slope between the exercise and non-exercise group. Secondary outcomes included IOP, MD at last visit, and visual acuity. Decimal visual acuity was converted to logarithm of the minimum angle of resolution (logMAR) for statistical analyses.

### Statistical analyses

Univariate comparisons were performed using the two-sided student’s *t*-test for continuous variables and the Fisher’s exact test for categorical variables. Prognostic factors for visual field defect progression were determined by logistic regression analysis. *P*-value ≤ 0.05 was considered statistically significant.

## Results

### Primary and secondary outcome measures

Of 24 patients who met the criteria, 11 and 13 patients were classified into the exercise and non-exercise groups, respectively. Patient backgrounds are summarized in Table 1. IOP, logMAR, and MD at the last visit were 14.8 ± 0.9 mmHg, 0.08 ± 0.04, and −8.22 ± 1.69 dB in the exercise group and 13.3 ± 0.8 mmHg, 0.08 ± 0.04 and −8.45 ± 1.55 dB in the non-exercise group. No significant differences in IOP (*P* = 0.24), logMAR (*P* = 0.93), or MD (*P* = 0.92) at the last visit were observed. The number of anti-glaucoma medications was 2.5 ± 0.4 and 2.2 ± 0.4 in the exercise and non-exercise groups, respectively. No significant differences were observed between the groups in the number of anti-glaucoma medications (*P* = 0.58).

**Table 1:** Patient backgrounds in the exercise group and non-exercise group

	Exercise group (*n* = 11)	Non-exercise group (*n* = 13)	*P*-value
Male/female	6/5	3/10	0.21^b^
Age (years)^a^	68.5 ± 10.9	69.1 ± 11.9	0.91^c^
IOP (mmHg)^a^	14.8 ± 0.9	13.3 ± 0.8	0.24^c^
LogMAR^a^	0.08 ± 0.04	0.08 ± 0.04	0.93^c^
MD (dB)^a^	−8.22 ± 1.69	−8.45 ± 1.55	0.92^c^
The number of anti-glaucoma drugs^a^	2.5 ± 0.4	2.2 ± 0.4	0.58^c^
Type of glaucoma			0.58^b^
Primary open-angle glaucoma	9 (81.8 %)	12 (92.3 %)	
Exfoliation glaucoma	2 (18.2 %)	1 (7.7 %)	

*MD* mean deviation, *LogMAR* logarithm of the minimum angle of resolution

^a^Mean ± standard deviation; ^b^Fisher’s exact test; ^c^Student’s t-test

MD slope in the exercise and non-exercise group was +0.20 ± 0.20 (mean ± standard error) dB/year and −0.53 ± 0.18 dB/year. Non-exercised group experienced faster visual field defect progression (*P* = 0.01). During the observational period, 8/11 (72.7 %) and 7/13 (53.8 %) patients experienced escalation of anti-glaucoma therapy. No significant differences were observed between the groups in escalation of anti-glaucoma therapy (*P* = 0.42; Table 2).

**Table 2:** Patient outcomes in the exercise group and non-exercise group in past 3 years

	Exercise group (*n* = 11)	Non-exercise group (*n* = 13)	*P*-value
MD slope (dB/year)^a^	0.20 ± 0.20	−0.53 ± 0.18	0.01*^b^
Escalation of anti-glaucoma therapy	8/11 (72.7 %)	7/13 (53.8 %)	0.42^c^

^*^ *P* < 0.05

^a^Mean ± standard error; ^b^Student’s *t*-test; ^c^Fisher’s exact test

### Prognostic factors for visual field defect progression

Age; sex; IOP; logMAR; MD; number of anti-glaucoma medications at last visit; escalation in anti-glaucoma therapy during the observational period; and self-reported exercise habits were evaluated as possible factors for visual field defect progression. In univariate logistic regression models (Table 3), two factors were statistically significant. Higher IOP and habitual exercise were found to be associated with visual field defect progression [odds ratio (OR) = 0.56; *P* = 0.003 per 1 mmHg increase and OR = 0.10; *P* = 0.01, respectively]. Significant predictors of visual field defect progression by multivariable logistic regression analysis (Table 3) were higher IOP (OR = 0.44 per 1 mmHg increase; *P* = 0.02) and habitual exercise (OR = 0.04; *P* = 0.02).

**Table 3:** Risk factors for visual field defect progression by logistic regression analyses

	Univariate		Multivariate	
	OR (95 % CI)	*P*-value	OR (95 % CI)	*P*-value
Age (years)^a^	0.98 (0.91–1.06)	0.62	1.06 (0.92–1.29)	0.41
Sex (M/F)	0.91 (0.17–4.88)	0.92	3.02 (0.19–104.7)	0.44
IOP (mmHg)^a^	0.56 (0.27–0.85)	0.003*	0.44 (0.11–0.92)	0.02*
LogMAR^a^	1.54 (0.001–1829.0)	0.90	192.4 (0.003–3.08 × 10^8^)	0.34
MD (dB)^a^	0.92 (0.77–1.07)	0.28	1.01 (0.67–1.45)	0.97
Habitual exercise	0.10 (0.01–0.59)	0.01*	0.04 (0.0003–0.70)	0.02*
Number of anti-glaucoma drugs^a^	0.66 (0.33–1.23)	0.20	0.46 (0.07–1.61)	0.23
Escalation of anti-glaucoma therapy	0.53 (0.09–2.83)	0.46	0.58 (0.02–14.36)	0.72

*OR* odds ratio, *CI* confidence interval, *IOP* intraocular pressure, *LogMAR* logarithm of the minimum angle of resolution, *MD* mean deviation

^*^ *P* < 0.05

^a^Per 1 mmHg increase

## Discussion

Exercise reduced IOP [4]. Changes in colloid osmotic pressure (a factor in capillary fluid exchange); increases in plasma osmolarity, ocular blood flow, and blood lactate; and decreases in blood pH have all been suggested as possible mechanisms that initiate a reduction in IOP. The exact mechanism is not elucidated. However, in this study, self-reported habitual exercise had no effect on IOP. This is probably due to small sample size. Glaucoma incidence was related to physical activity in a cohort of 29,854 male runners [5]. However, no other studies showed a relationship between exercise and glaucomatous visual field defect progression in glaucoma patients. Thus, we believe that this study is the first to demonstrate a relationship between exercise and visual field loss progression in glaucoma patients.

Here high IOP was related to slower visual field loss progression, which is in contrast with previous reports [7]. In clinical settings, patients with rapidly advanced visual field defect tend to receive more intensive treatments with lower IOP targets. This clinical decision may contribute to lower IOP in progressing patients. Prospective and large-scale studies are required to fully elucidate the relationship between IOP and visual field loss progression. Although the mechanism underlying the effect of exercise in deteriorating visual field loss progression remains unknown, exercise was shown to be beneficial for vascular profiles [8]: The smaller preresistance and resistance vessels are involved in younger individuals, whereas the large elastic arteries are involved in older individuals. Active individuals had a lower risk of low ocular perfusion pressure [9]. These studies indicate that exercise may affect disc circulation and attenuate visual field loss.

This study had several limitations. It was a retrospective, single-centered, small sample size study. We evaluated MD slopes to analyze visual field defect progression. Different results may have been obtained with visual field index or point-wise event analysis.

## Conclusion

This study suggested that self-reported habitual exercise is associated with slower visual field progression in open-angle glaucoma patients. It is necessary for larger scale, and prospective cohort study to clarify the relationship between exercise and glaucomatous visual field defects. Even though, it may be clinically beneficial to encourage glaucoma patients to have habitual exercise.

## Additional file

Additional file 1: Analyzed datasets. (XLSX 36 kb)

## Abbreviations

HFA: Humphrey field analyzer
IOP: Intraocular pressure
logMAR: Logarithm of the minimum angle of resolution
MD: Mean deviation
OR: Odds ratio
SITA: Swedish interactive thresholding algorithm
